# Mice expressing a “hyper-sensitive” form of the CB_1_ cannabinoid receptor (CB_1_) show modestly enhanced alcohol preference and consumption

**DOI:** 10.1371/journal.pone.0174826

**Published:** 2017-04-20

**Authors:** David J. Marcus, Angela N. Henderson-Redmond, Maciej Gonek, Michael L. Zee, Jill C. Farnsworth, Randa A. Amin, Mary-Jeanette Andrews, Brian J. Davis, Ken Mackie, Daniel J. Morgan

**Affiliations:** 1 Department of Psychological and Brain Sciences and The Linda and Jack Gill Center for Biomolecular Science, Indiana University, Bloomington, IN, United States of America; 2 Department of Anesthesiology, Penn State University College of Medicine, Hershey, PA, United States of America; Nathan S Kline Institute, UNITED STATES

## Abstract

We recently characterized S426A/S430A mutant mice expressing a desensitization-resistant form of the CB_1_ receptor. These mice display an enhanced response to endocannabinoids and ∆^9^-THC. In this study, S426A/S430A mutants were used as a novel model to test whether ethanol consumption, morphine dependence, and reward for these drugs are potentiated in mice with a “hyper-sensitive” form of CB_1_. Using an unlimited-access, two-bottle choice, voluntary drinking paradigm, S426A/S430A mutants exhibit modestly increased intake and preference for low (6%) but not higher concentrations of ethanol. S426A/S430A mutants and wild-type mice show similar taste preference for sucrose and quinine, exhibit normal sensitivity to the hypothermic and ataxic effects of ethanol, and have normal blood ethanol concentrations following administration of ethanol. S426A/S430A mutants develop robust conditioned place preference for ethanol (2 g/kg), morphine (10 mg/kg), and cocaine (10 mg/kg), demonstrating that drug reward is not changed in S426A/S430A mutants. Precipitated morphine withdrawal is also unchanged in opioid-dependent S426A/S430A mutant mice. Although ethanol consumption is modestly changed by enhanced CB_1_ signaling, reward, tolerance, and acute sensitivity to ethanol and morphine are normal in this model.

## Introduction

Alcohol and morphine use disorders are highly prevalent, affecting an estimated 16.6 million [1] individuals worldwide and 2.1 million Americans [2], respectively. In 2010, 16,651 deaths in the United States were attributed to overdose from heroin and prescription opioids [3]. Abuse of these drugs inflicts a tremendous financial burden on society, with alcohol dependence alone costing in excess of $223.5 billion annually [4,5].

The relative lack of long-term efficacy for currently available treatments for alcohol and opiate use disoders illustrates the need for additional therapeutic agents for treating addiction to these drugs [6–10]. Preclinical rodent models have shown that genetic and pharmacological inhibition of the endocannabinoid signaling system (ECS) can profoundly impact ethanol- [11] and opioid- [12,13] motivated behaviors and the physiological responses to these drugs. Treatment with morphine and ethanol cause dopamine release in the nucleus accumbens [14–18]. Mice lacking CB_1_ lack ethanol-induced dopamine release in the nucleus accumbens [19,20]. Inhibition of the ECS with rimonabant, a CB_1_ inverse agonist, blocks ethanol- but not heroin-stimulated dopamine signaling in the nucleus accumbens [17,21,22]. Conditioned place preference (CPP) for ethanol and morphine are absent in CB_1_ knock-out mice [20,23–26]. Dependence on ethanol and opiates is also attenuated in CB_1_ deficient mice and rodents treated with rimonabant [24,25,27–29]. Thus, understanding the precise mechanisms through which alcohol influences the ECS and vice versa has the potential to positively impact treatment of alcohol and opioid use disorders.

The ECS consists of three main components: the endogenous ligands (endocannabinoids), the cannabinoid receptors, and the enzymes responsible for the synthesis and catabolism of endocannabinoids. Two main endocannabinoids have been identified, N-arachidonoyl ethanolamine (AEA; anandamide) [30] and 2-arachidonoyl-glycerol (2-AG) [31,32]. CB_1_ mediates the psychoactive effects of delta-9-tetrahydrocannabinol (∆^9^-THC) and is widely expressed throughout the central nervous system [33–38].

Although mice lacking CB_1_ clearly show attenuated morphine and ethanol motivated behaviors, it is not well understood whether enhanced activation of the ECS facilitates these behaviors. This study was performed to test the hypothesis that enhanced CB_1_ signaling potentiates opiate and alcohol addiction in a preclinical mouse model. These experiments used recently characterized “knock-in” mice expressing a desensitization-resistant form of CB_1_ (S426A/S430A) that exhibit markedly enhanced acute responses to endocannabinoids and exogenously administered ∆^9^-THC [39]. First, we examined voluntary ethanol drinking using an unlimited-access, two-bottle choice assay to determine whether S426A/S430A mutant mice consume more ethanol than wild-type controls. Second, conditioned place preference (CPP) assays were performed to measure whether the rewarding properties of morphine, ethanol, or cocaine were potentiated in S426A/S430A mutant mice. Third, we assessed sensitivity to the ataxic and hypothermic effects of ethanol and the recovery of ethanol-induced loss of righting reflex to determine whether a decrease in senstivity to ethanol was responsible for the increased consumption of ethanol we observed for the S426A/S430A mutants. Fourth, we measured tolerance to the antinociceptive and hypothermic effects of daily morphine injections. Finally, we scored precipitated withdrawal from morphine to determine whether morphine dependence was potentiated in S426A/S430A mutants. The results of this study show that low concentration ethanol consumption is modestly increased in S426A/S430A mutants. However, reward for ethanol, morphine, and cocaine as well as sensitivity and tolerance to ethanol and morphine are unaltered. Thus, our findings suggest that elevated ECS signaling due to disruption of desensitization has only a modest effect on ethanol drinking and that the utility of S426A/S430A mutants for addiction research is limited.

## Materials and methods

### Subjects

Experiments were performed using 8–16 week old male age-matched littermate S426A/S430A mutant and wild-type mice. All mice used were either homozygous for the S426A/S430A mutation or homozygous wild-type mice on a C57Bl6/129SvEv hybrid background. Mice used in this study were backcrossed to the C57Bl6 strain for 2–4 generations (F_2_-F_4_ backcrosses). Mice used in these experiments were individually (drinking and morphine dependence experiments) or group (all other experiments) housed under a 12:12 h light–dark cycle (lights on 07:00, lights off 19:00) and provided with standard mouse chow ad libitum. All animal care and experimental procedures used in this study were approved by the Institutional Animal Care and Use Committee of the Penn State University College of Medicine or Indiana University Bloomington and conform to the Guidelines of the National Institutes of Health on the Care and Use of Animals.

### Drugs

A 100% ethanol stock solution was diluted to 20% (v/v) in 0.9% physiological saline. A dose of 1 or 2 g/kg was administered intraperitoneally (IP) in an injection volume of 12.5 ml/kg for experiments on ethanol CPP. To assess ethanol sensitivity, 100% ethanol stock was diluted to either 20 or 30% (v/v) in 0.9% saline and administered IP in doses of 0, 1, 1.5, 2.0, 2.5, 3.0, and 3.5 g/kg. To assess recovery from the sedative effects of ethanol in the loss of righting reflex, 100% ethanol stock was diluted to 25% in 0.9% sterile saline and given by IP injection at a dose of 4 g/kg. For the two-bottle choice drinking assay, ethanol was diluted in tap water to concentrations of 3, 6, 9, 12, 15, and 18% (v/v). Sucrose (Fisher, Pittsburgh, PA) was dissolved into tap water for final concentrations of 1.7 and 4.25% (w/v) while quinine hydrochloride (Avantor, Center Valley, PA) was dissolved to reach final concentrations of 0.03mM and 0.1mM. Cocaine hydrochloride (NIDA Drug Supply, Bethesda, MD) was dissolved in 0.9% saline and given at a dose of 10 mg/kg IP in an injection volume of 10 ml/kg. Morphine sulfate (NIDA Drug Supply, Bethesda, MD) was dissolved in 0.9% saline and administered subcutaneously (SC) at a dose of 10 mg/kg in an injection volume of 10 ml/kg. A 75mg morphine pellet (NIDA Drug Supply, Bethesda, MD) was implanted subcutaneously to induce morphine dependence. Naloxone hydrochloride (Cayman, Ann Arbor, MI) was dissolved in 0.9% saline and 1 mg/kg was injected IP in a volume of 10 ml/kg to precipitate morphine withdrawal.

### Two-bottle choice drinking

Ethanol consumption and preference were measured in both male S426A/S430A and wild-type littermates using a modified version of the two-bottle choice assay previously described [40]. Briefly, mice were individually housed and habituated in cages containing two water bottles for six days. Following habituation, one of the water bottles was replaced with a bottle containing 3% ethanol, and mice were given unlimited access to both bottles. Body weights and fluid intakes were recorded and the bottle positions were alternated every other day to control for the development of a side preference. Every eight days, the concentration of ethanol was increased (3%, 6%, 9%, 12%, 15%, and 18% ethanol v/v). Ethanol intake was calculated in grams of ethanol per kilogram (g/kg) of body weight per day and ethanol preference as the volume of ethanol consumed as a percentage of the total fluid consumed. Using this same two-bottle choice paradigm, S426A/S430A mutant and wild-type mice were also assessed for any differences in their taste preference or aversion to 1.7% and 4.25% sucrose (w/v) and 0.03mM and 0.1mM quinine. In all preference and intake studies, four empty cages with water and ethanol bottles were used as controls for spillage and solution evaporation.

### Conditioned place preference (CPP)

CPP for ethanol (1 and 2 g/kg) was assessed in 31 S426A/S430A mutant and 30 wild-type littermates (14–16 mice/group). Mice were tested in three-chamber place-conditioning boxes (Med Associates, St. Albans, VT) that were housed in sound-attenuating chambers. CPP experiments for ethanol were done using three-chamber place conditioning boxes and was performed after Dr. Morgan moved from Indiana University to start his own laboratory at the Penn State University College of Medicine. The experiment consisted of four phases: habituation (one session), baseline preference (one session), conditioning (eight sessions) and preference testing (one session). Sessions were scheduled over 11 consecutive days. During the habituation session (Day 1), mice were given 30 minutes to explore all sections of the CPP apparatus to reduce any novelty effects. The following day (Day 2), mice were given 30 minutes of access to the CPP apparatus during which they could explore all chambers to determine their baseline preferences (pre-conditioning scores). Following the baseline preference session, mice were randomly assigned to have ethanol (CS^+^) or saline (CS^-^) paired with either the black or white conditioning chambers. During the conditioning phase (Days 3–10), mice were injected with either saline or ethanol (1 or 2 g/kg, IP) on alternating days (i.e., days 3,5,7,9 or 4,6,8,10). Immediately following an injection, mice were confined to the appropriate conditioning chamber for 5 minutes, an optimal amount of time for ethanol place preference conditioning. The order of saline (CS^-^) and ethanol (CS^+^) exposure was counterbalanced within groups. After eight total conditioning sessions (4 ethanol and 4 saline), mice were given a 30 minute preference testing session (Day 11) without any drug on board. The amount of time spent in each chamber was recorded and a CPP was determined by assessing the amount of time (in seconds) mice spent in the drug-paired chamber pre- versus post-conditioning.

Separate groups of S426A/S430A mutant and littermate mice were also assessed for CPP to 10 mg/kg of cocaine (IP) and 10 mg/kg of morphine (SC). Although a similar testing paradigm was used [11 days; habituation (1 day), pre-test (1 day), condition (8 days), post-test (1 day)], mice in these experiments were tested at Indiana University in 2 (versus 3) chambered place conditioning boxes (Accuscan Instruments, Columbus, OH) and conditioning sessions lasted for 30 (vs. 5) minutes, an optimal conditioning session length for cocaine place preference. As in the ethanol experiment, the CS^+^ tactile and visual environment was randomly assigned in an unbiased, counter-balanced subject assignment scheme as previously described [41]. As with ethanol, the development of a CPP was determined by assessing the amount of time (in seconds) that mice spent in the drug (CS^+^)-paired chamber in pre- versus post-conditioning test sessions.

### Ethanol sensitivity

Sensitivity to the hypothermic and ataxic effects of ethanol was examined in 11 wild-type and 19 S426A/S430A mutant mice. Ethanol-induced ataxia was assessed using a rotarod (Med-Associates, St. Albans, VT) set to accelerate from 4 to 40 rpm at a constant rate over 300 seconds. Mice were given six training trials on the rotarod the day prior to ethanol sensitivity training. On testing days, ethanol-induced ataxia was measured in duplicate 30 minutes after injection of ethanol (IP). Rectal temperatures (Physitemp, Clifton, NJ) were taken prior to ethanol administration, and hypothermia was assessed at 30, 60, 120, and 180 minutes post-injection. The effects of 0, 1, 1.5, 2, 2.5, 3, and 3.5 grams per kilogram (g/kg) of ethanol were examined in a single cohort of mice. Mice were allowed at least seven days of recovery between each dose of ethanol, and doses were tested from lowest to highest in order to minimize the effects of tolerance. Sensitivity to the sedative effects of ethanol was also assessed using the loss of righting reflex in 8 wild-type and 7 S426A/S430A mutant mice. Mice were injected with 4 g/kg of ethanol. Following loss of righting reflex (typically in under two minutes), mice were placed upside down in an inverted v-shaped trough so that all four paws were up in the air. Mice were measured for the amount of time (in minutes) it took for them to right themselves. Regaining of the righting reflex was defined as the mouse being able to successfully right itself 3 times within 30 seconds.

### Blood ethanol concentration (BECs)

BECs for 9–11 wild-type and 7–8 S426A/S430A mutant mice were assessed at 15, 30, 60 and 120 minutes following a bolus IP injection of 2 g/kg of ethanol. Blood samples (approximately 50 μl) were collected from lateral tail vein nicks using microhematocrit capillary tubes (Fisher Scientific, Pittsburg, PA). Plasma was isolated by centrifugation at 10,000 x g for 10 minutes, and BECs (mg/dL) were assayed using an Analox alcohol analyzer (Analox Instruments, Lunenberg, MA).

### Morphine tolerance

S426A/S430A mutant and wild-type littermates were assessed daily for tolerance to the antinociceptive and hypothermic effects of morphine. Mice were injected once daily with 10 mg/kg morphine (SC) for 10 consecutive days. Antinociception was measured using a Columbus Instruments TF-1 tail-flick analgesia meter (Columbus, OH) and a Columbus Instruments Hot Plate Analgesia Meter (Columbus, OH). The heat source was calibrated to elicit a tail-flick latency of 3–4 seconds (intensity setting of 5) in untreated wild-type mice and the hotplate was set at 55°C. Cutoffs of 10s and 30s for tail-flick and hotplate testing sessions, respectively, were used to avoid tissue damage to the tail and/or paws. Tail-flick and hotplate latencies were measured immediately before morphine treatment and 60 minutes afterwards to calculate the antinociceptive responses as the percentage of maximal possible effect (%MPE) with %MPE = [(post-morphine latency)–(pre-morphine latency)]/[10-(pre-morphine latency)] x 100 for tail-flick and %MPE = [(post-morphine latency)–(pre-morphine latency)]/[30-(pre-morphine latency)] x 100 for the hotplate assay. Hypothermia was assessed by taking body temperatures using a mouse rectal thermometer (Physitemp Instruments, Clifton, NJ), immediately prior to and 60 minutes after morphine injection and was recorded as percent change in body temperature (% ∆BT) = [(pre-morphine temperature)–(post-morphine temperature)]/[pre-morphine temperature] x 100.

### Morphine dependence

The severity of morphine dependence was evaluated by scoring various symptoms of naloxone-precipitated morphine withdrawal. Mice were anesthetized using a tabletop laboratory animal anesthesia system (Vetequip Incorporated, Livermore, CA) delivering 4% isoflurane for induction and 2.5% isoflurane for maintenance of anesthesia. Once anesthetized, a two-inch diameter circle was shaved on the neck/back of the mouse. The surgical site was further prepared by cleaning with chlorohexadine, iodine, and then ethanol. Using a scalpel, a 0.25-inch subcutaneous (SC) incision was made perpendicular to the length of the mouse just below the nape of the neck. Using hemostatic forceps, a 75mg morphine pellet (NIDA Drug Supply, Bethesda, MD) was implanted into the pouch created from the incision. The incision was closed with two wound clips, and the mice were then removed from isoflurane and allowed to recover on a heating pad. Seventy-two hours post-implantation, mice received a 1 mg/kg IP injection of naloxone (Cayman, Ann Arbor, MI) to precipitate withdrawal. Immediately after the injection, mice were placed into a transparent plastic enclosure so that a web camera could record precipitated withdrawal for one hour. Jumps, paw tremors, wet dog shakes, and diarrhea were scored from the videos by a single trained and blinded observer.

### Data analysis

Two-way mixed factorial analysis of variance (ANOVAs) in Prism 6 (Graphpad, La Jolla, CA) was used to analyze ethanol drinking, sucrose and quinine preference, CPP, morphine tolerance, and ethanol sensitivity with genotype as the between-subjects factor and day/dose/or conditioning as the within-subjects factor. During the two-bottle ethanol choice assay, two-way mixed ANOVAs were run between genotype across the eight days of drinking recorded within each ethanol concentration tested and the averages were then plotted. BECs were analyzed using a two-way ANOVA as different groups of mice were used for each dose. For all ANOVAs, Bonferroni post hoc tests were performed where appropriate and in all analyses significance was set at *p*<0.05. The total number of precipitated withdrawal symptoms and loss of righting reflex was analyzed using Prism 6 to perform Student’s t tests.

## Results

### Two-bottle choice drinking

**Fig 1:**
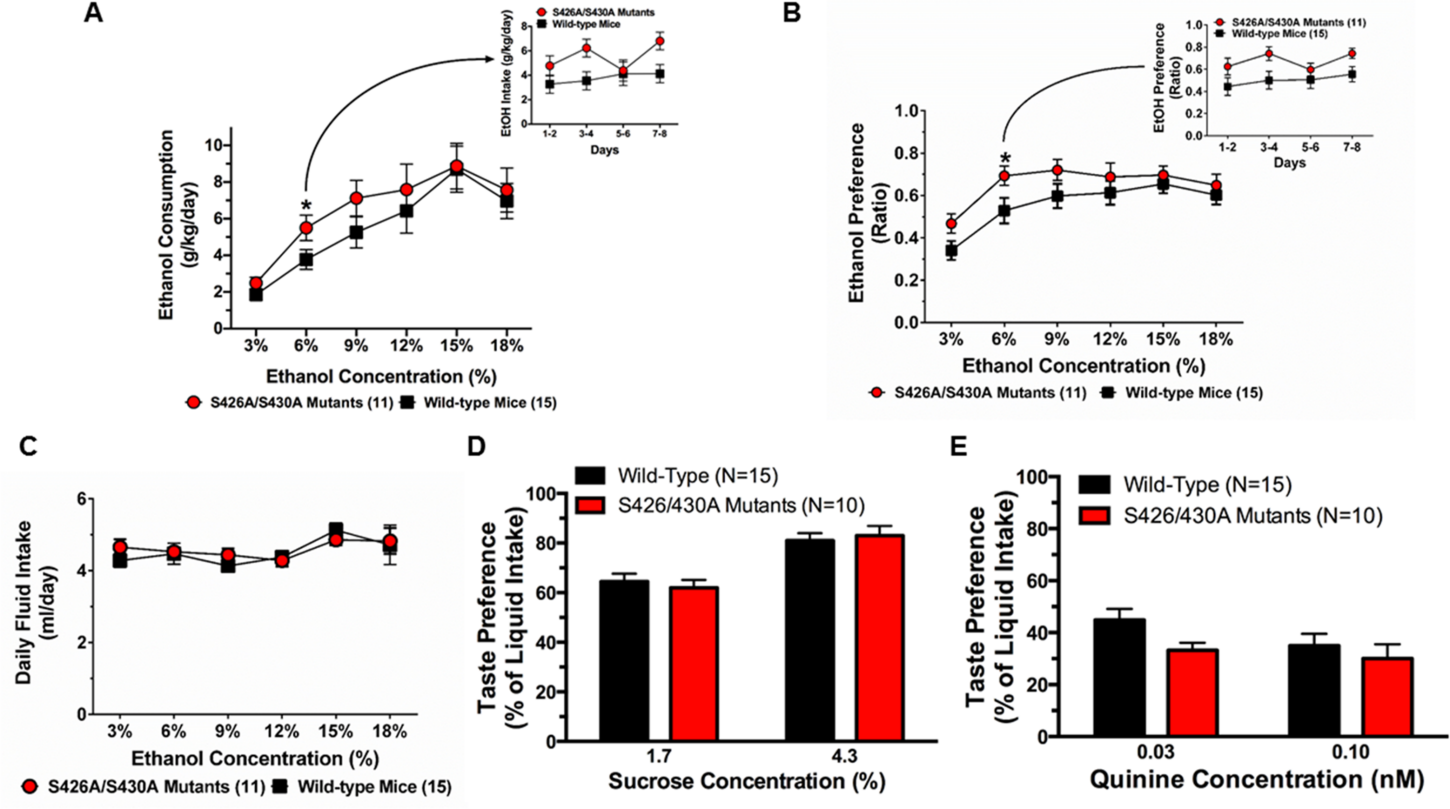
S426A/S430A mutants prefer and consume more ethanol. **A**. Consumption (g/kg) of a low (6%) concentration of ethanol was increased for S426A/S430A mutants (red circles and line) relative to wild-type littermates (black lines and squares). Mice were given 24 hour unlimited access to 3%, 6%, 9%, 12%, 15%, and 18% ethanol (v/v) using a two-bottle choice assay. **B**. S426A/S430A mutants also prefer 6% ethanol more than wild-type littermates. **C**. Total liquid intake (water plus ethanol) was identical for S426A/S430A mutants and wild-type mice. **D**. Consumption of 1.75% and 4.25% sucrose was equivalent for S426A/S430A mutants (red bars) and wild-type littermates (black bars). **E**. S426A/S430A mutants (red bars) and wild-type littermates (black bars) also consume similar amounts of 0.03 mM and 0.1 mM quinine solutions. Averages across the 8 days of intake are shown for each concentration; for both **A** and **B**, intakes (collected every 48 hours) are shown in the inset for 6% ethanol. Error bars represent the SEM and data analyses were performed using two-way repeated-measure ANOVA with Bonferroni post-hoc tests (*p<0.05). Sample sizes for each group are in parentheses.

Voluntary ethanol consumption and preference were measured in S426A/S430A mutants and wild-type mice given unlimited 24 hour ethanol access using a two-bottle choice assay. Results from individual repeated measure ANOVAs found a main effect of genotype at 6% (*F*_1,24_ = 4.332, *p* = 0.0482) only. There were no main effects of genotype found at 3% (*p* = 0.1477), 9% (*p* = 0.1629), 12% (*p* = 0.3661), 15% (*p* = 0.7294) or 18% (*p* = 0.5623) ethanol. S426A/S430A mutant mice showed increased consumption (Fig 1A) and preference (Fig 1B) (*F*_1,24_ = 5.088, *p* = 0.0335) for 6% ethanol. The increase in consumption and preference for 6% ethanol was consistent across each of the four (48 hour) measurement sessions (Fig 1A and 1B insets). The lack of any changes in consumption and preference for 15% was also consistent across each measurement session (S1 and S2 Figs). We found that the total amount of liquid consumed (ethanol and water) was virtually identical for S426A/S430A mutant and wild-type mice (Fig 1C).

Taste preference for sweet- and bitter-flavored solutions was also examined using a two-bottle choice assay to determine whether altered taste preference was responsible for the increased ethanol preference and consumption in the S426A/S430A mutant mice. S426A/S430A mutant and wild-type mice displayed identical taste preference for 1.75% and 4.25% sucrose (Fig 1D). S426A/S430A mutant mice exhibited nearly equivalent taste aversion for 0.03mM and 0.10mM quinine compared to wild-type littermates (Fig 1E).

### Ethanol conditioned place preference

**Fig 2:**
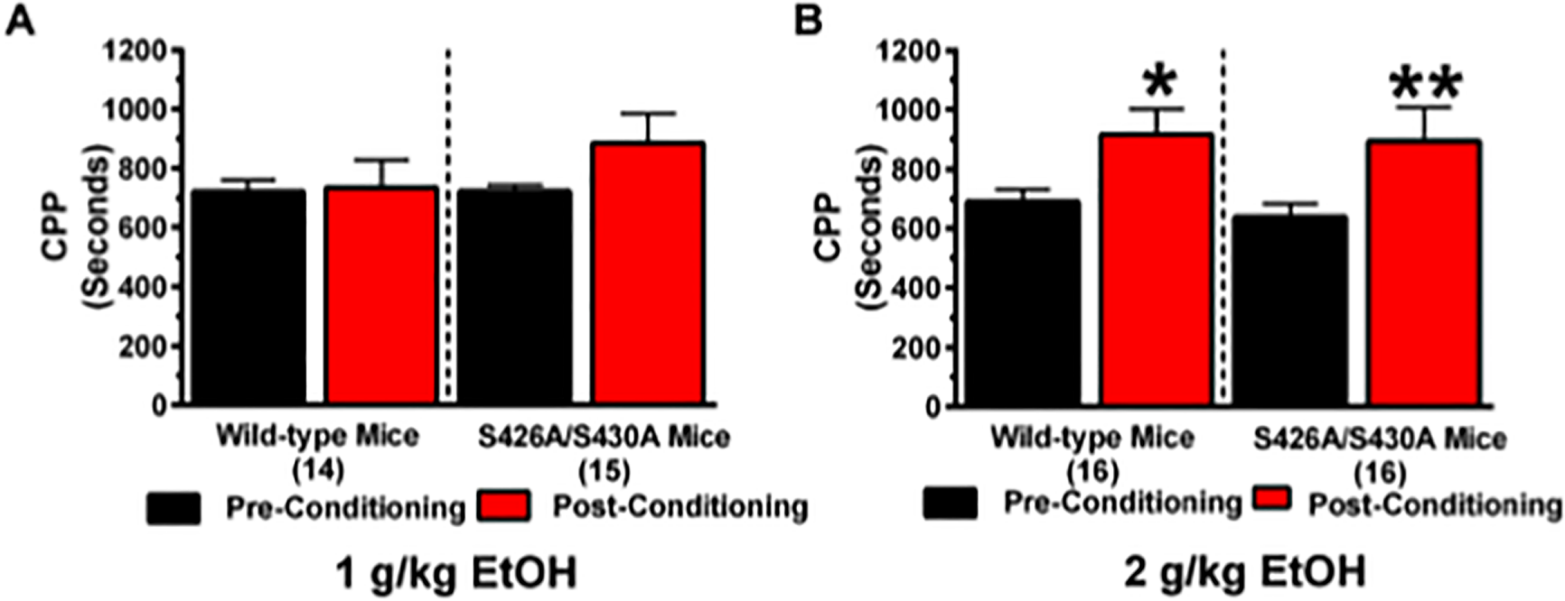
S426A/S430A mutants show normal conditioned place preference for ethanol. CPP for 1 and 2 g/kg of ethanol was examined in S426A/S430A mutants and wild-type littermates. Pre-conditioning (black bars) and post-conditioning (red bars) place preference was measured before and after eight days of place preference conditioning for (**A**) 1 and (**B**) 2 g/kg of ethanol. Error bars represent the SEM and data analyses were performed using a two-way repeated measures ANOVAs with Bonferroni post-hoc tests (*p<0.05). Sample sizes for each group are in parentheses.

CPP for 1 and 2 g/kg of ethanol was performed to investigate whether the rewarding effects of ethanol were increased for the S426A/S430A mutant mice. We hypothesized that ethanol CPP would be stronger in S426A/S430A mutants. While there was no effect of conditioning at 1 g/kg of ethanol (Fig 1A) (*F*_1,27_ = 1.982; *p* = 0.1706), mice displayed a positive effect of conditioning as evidenced by robust yet equivalent CPP for 2 g/kg of ethanol (Fig 2B) (*F*_1,30_ = 17.71; *p* = 0.0002). However, no effect of genotype or a genotype x conditioning interaction was detected.

### Ethanol sensitivity

**Fig 3:**
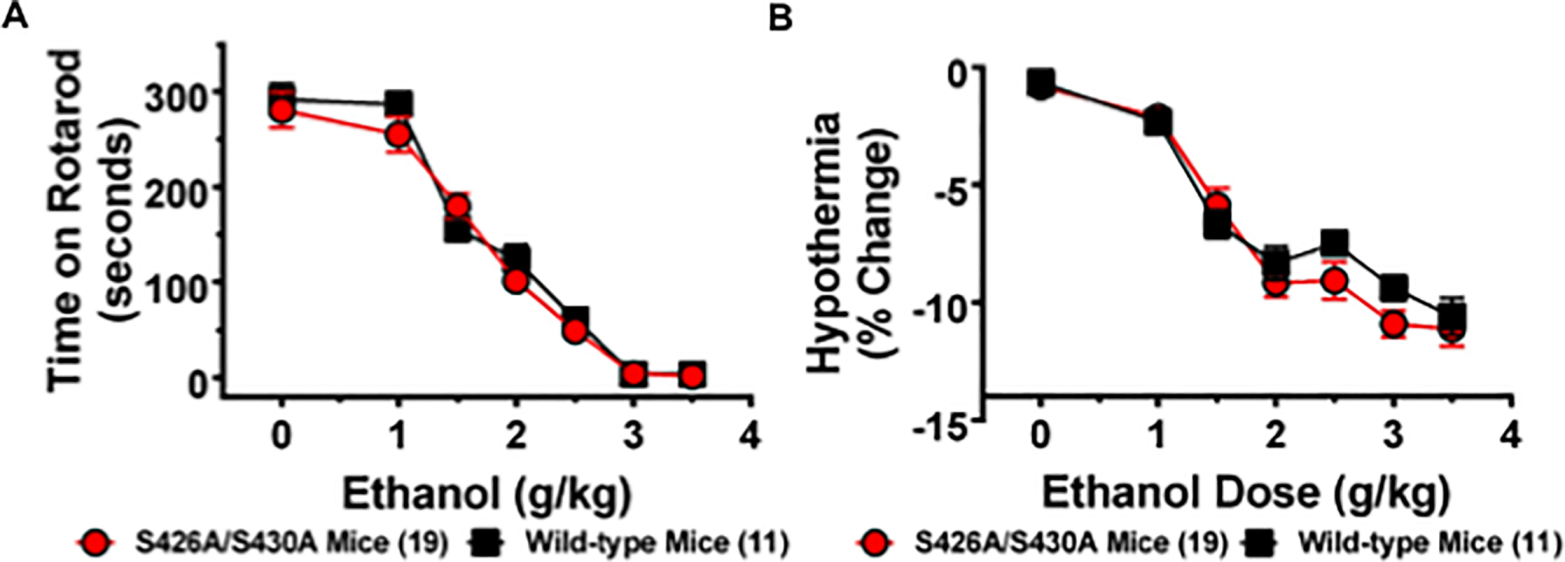
Sensitivity to ethanol is not altered for S426A/S430A mutants. **A.** The ataxic effects of 0 (saline only), 1, 1.5, 2, 2.5, 3, and 3.5 g/kg of ethanol were measured in S426A/S430A mutants (red circles and line) and wild-type littermates (black lines and squares) using an accelerating rotarod. S426A/S430A mutant mice show the same sensitivity to the ataxic effects of ethanol as wild-type mice. **B**. S426A/S430A mutants also display the same sensitivity to the hypothermic effect of ethanol as wild-type mice. Error bars represent the SEM and data analyses were performed using two-way repeated measure ANOVAs with Bonferroni post-hoc tests. Sample sizes for each group are in parentheses.

**Fig 4:**
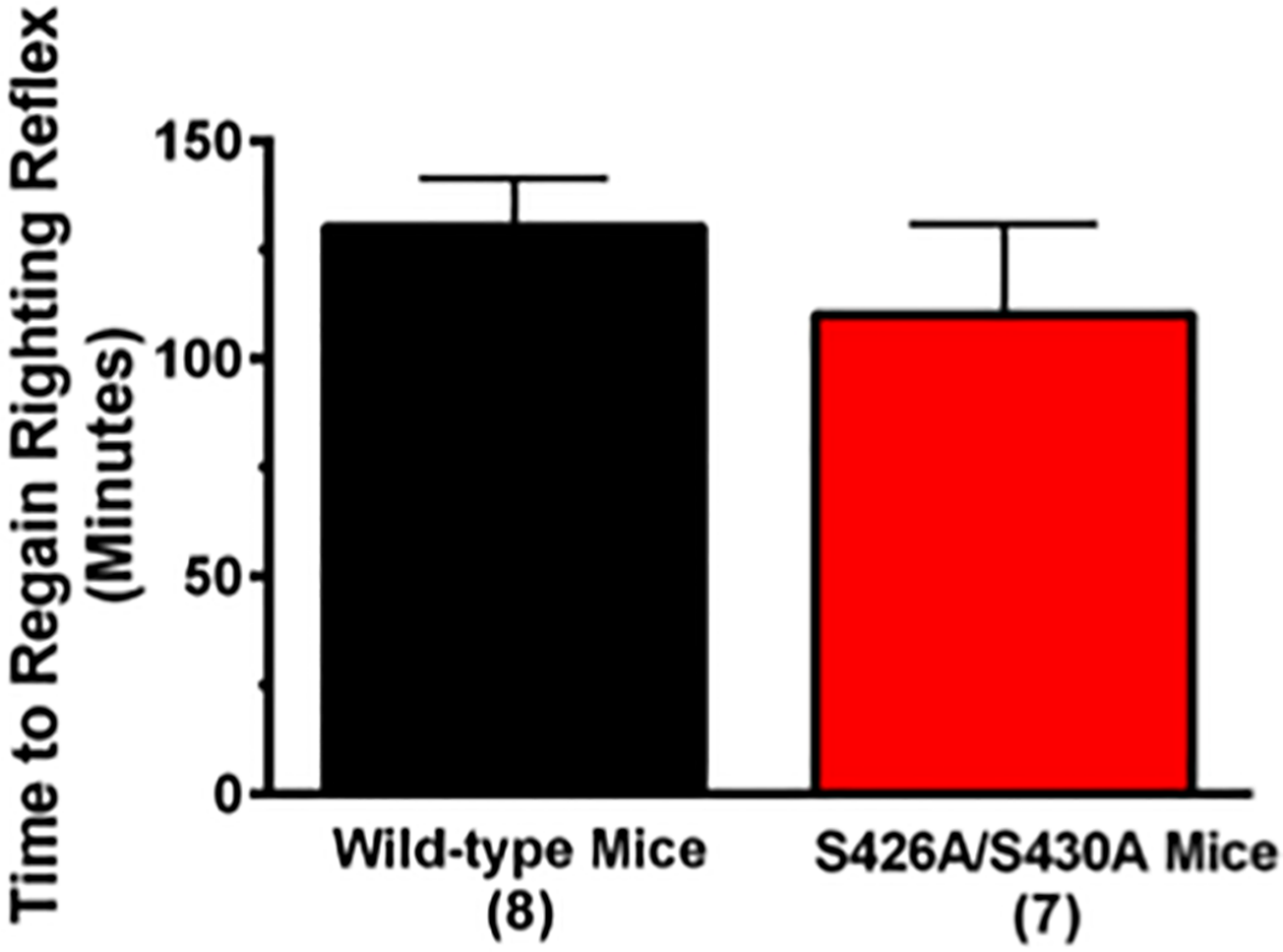
Time to recover the righting reflex is not altered in S426A/S430A mutants. The amount of time to regain the righting reflex was measured following administration of an ataxic dose (4 g/kg) of ethanol in both S426A/S430A (red bar) and wild-type (black bar) littermates via the loss of righting reflex assay. Error bars represent the SEM and data analysis was done using a t-test. Sample sizes for each group are in parentheses.

The acute hypothermic and ataxic effects of ethanol were measured to test the hypothesis that decreased sensitivity to ethanol might drive the increased ethanol consumption observed in the S426A/S430A mutant mice (Fig 1). This possibility was examined by generating a dose-response curve measuring the ataxic and hypothermic effects of 0 (saline), 1, 1.5, 2, 2.5, 3, and 3.5 g/kg of ethanol in S426A/S430A mutant and wild-type mice. Both S426A/S430A mutant and wild-type mice exhibited a dose-dependent increase in ethanol-induced ataxia (Fig 3A) (*F*_6,168_ = 121.5; *p*< 0.0001) that was not different between mutants and wild-type mice (*p* = 0.97). Similar to ataxia, S426A/S430A mutant and wild-type mice also exhibited a dose dependent increase in ethanol-induced hypothermia (Fig 3B) (*F*_6,168_ = 60.82; *p*<0.0001) that was not different between mutants and wild-type mice (*p* = 0.38). No dose x genotype interaction effects were detected for either ethanol-induced ataxia (*p* = 0.90) or hypothermia (*p* = 0.22). Recovery of the righting reflex was also measured to assess the magnitude of ethanol-induced sedation in S426A/S430A mutant mice compared to wild-type littermates. Student’s t-test revealed that there was no difference in the amount of time it took for wild-type and S426A/S430A mutant mice to recover their righting reflexes following a challenge dose of 4 g/kg of ethanol (Fig 4) (*t*_13_ = 0.890; *p* = 0.390).

### Ethanol metabolism

**Fig 5:**
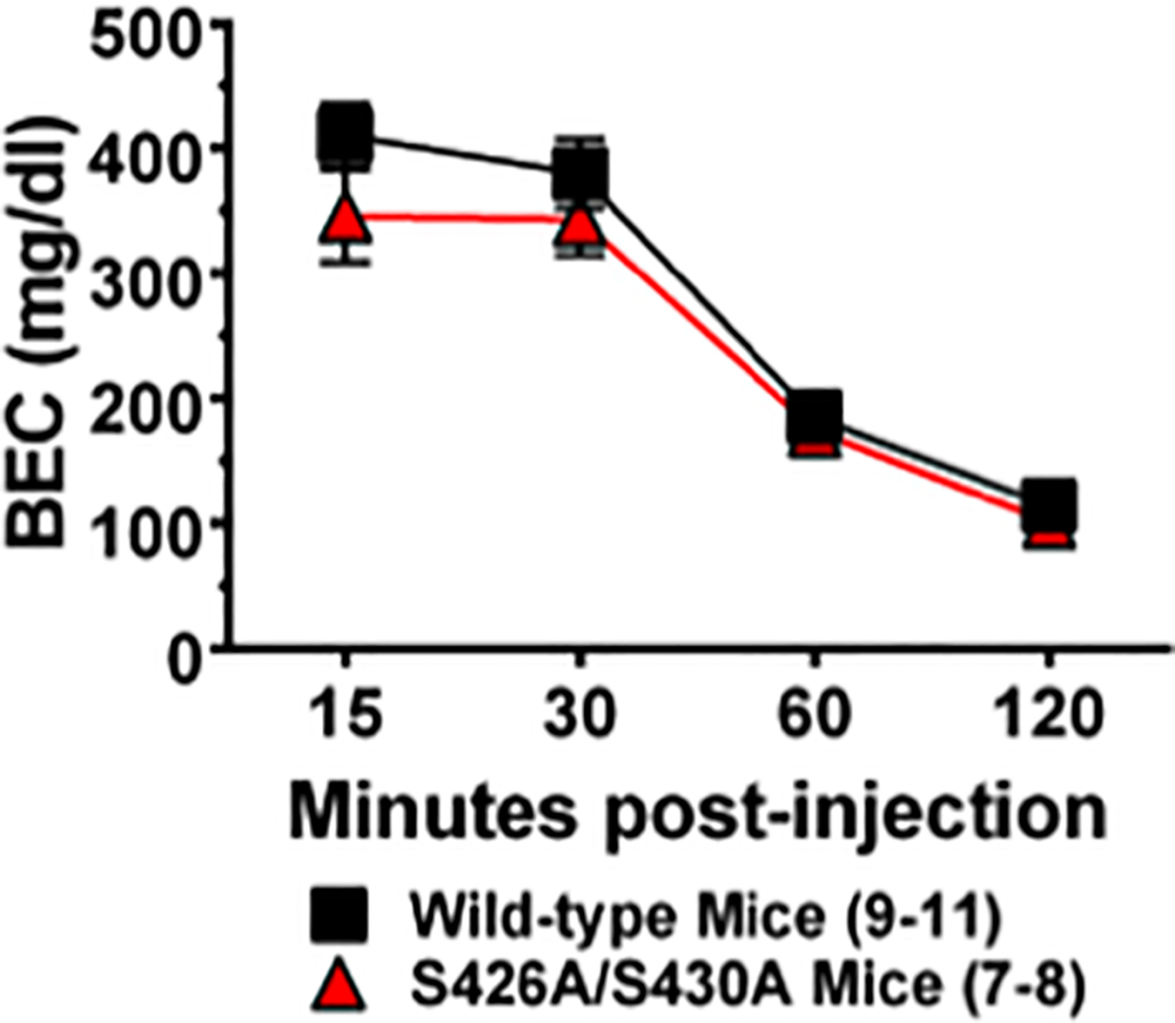
Ethanol metabolism is not altered in S426A/S430A mutants. Blood ethanol concentrations were measured in S426A/S430A mutants (red bars) and wild-type littermates (black bars) 15, 30, 60 and 120 minutes after a bolus injection (IP) of 2 g/kg of ethanol. Error bars represent the SEM and data analyses were performed using two-way ANOVA with Bonferroni post-hoc tests. Sample sizes for each group are in parentheses.

BECs were measured 15, 30, 60 and 120 minutes following a bolus injection of 2 g/kg (IP) of ethanol in order to determine whether more rapid ethanol metabolism might underlie increased ethanol drinking in S426A/S430A mice. S426A/S430A mutant and wild-type mice exhibited time-dependent metabolism of ethanol (decrease in BEC) (Fig 5) (*F*_3,62_ = 89.91; *p*<0.0001). While no time x genotype interaction effect was detected (*p* = 0.77), there was a slight overall effect of genotype on ethanol metabolism (*F*_1,62_ = 4.825, *p* = 0.0318). However, post hoc comparisons failed to yield any genotype differences at any of the time points tested.

### Morphine and cocaine conditioned place preference

**Fig 6:**
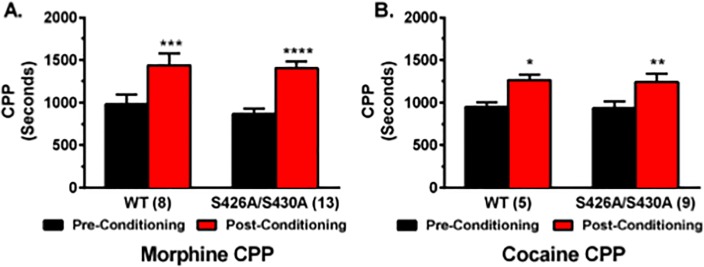
Conditioned place preference for morphine and cocaine is not changed in S426A/S430A mutants. CPP for either 10 g/kg of morphine (**A**) or 10 mg/kg cocaine (**B**) was examined in S426A/S430A mutants and wild-type littermates. Pre-conditioning (black bars) and post-conditioning (red bars) place preference was measured before and after eight days of place preference conditioning. Error bars represent the SEM and data analyses were performed using two-way repeated measure ANOVAs with Bonferroni post-hoc tests (*p<0.05, **p<0.01, ***p<0.001, ****p<0.0001). Sample sizes for each group are in parentheses.

CPP for 10 mg/kg of cocaine and 10 mg/kg morphine were investigated to determine whether the rewarding effects of other drugs of abuse were affected by the S426A/S430A mutation. Cocaine, in particular, was selected as a positive control because it is strongly reinforcing in the CPP paradigm and also because previous work has demonstrated that the ECS is not involved in cocaine CPP [24]. These experiments examining cocaine and morphine CPP were performed using two-chamber CPP boxes (Indiana University) instead of three-chamber CPP boxes (Penn State University). Similar to ethanol, mice collectively displayed a strong CPP for 10 mg/kg morphine (Fig 6A) (*F*_1,19_ = 39.57; *p*<0.0001 with no effect of genotype (*p* = 0.53) or any interaction effect (*p* = 0.55). Mice also displayed a strong CPP for 10 mg/kg cocaine (Fig 6B) (*F*_1,12_ = 19.32; *p* = 0.0009) with no interaction (*p* = 0.96) or effect of genotype (*p* = 0.86).

### Morphine tolerance

**Fig 7:**
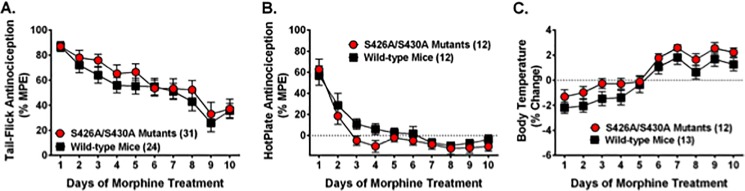
Tolerance to the antinociceptive and hypothermic effects of morphine is normal in S426A/S430A mutant mice. Tolerance to the antinociceptive and hypothermic effects of daily treatment with morphine were examined in S426A/S430A mutant mice (red line and circles) and wild-type littermates (black line and squares). Tail-flick antinociception **(A)**, hotplate antinociception **(B)**, and body temperature **(C)** were measured 60 minutes after SC injection of 10 mg/kg morphine. Data are expressed as mean ± S.E.M and sample sizes for each group are in parentheses. Data analyses were performed using two-way repeated measure ANOVAs with Bonferroni post-hoc tests.

Tolerance to the antinociceptive and hypothermic effects of 10 mg/kg morphine was compared between S426A/S430A mutant and wild-type mice (Fig 7). There was a significant effect of time on the development of morphine tolerance using the tail-flick test to assess antinociception (Fig 7A) (*F*_9,477_ = 18.98; *p*<0.0001), but there was no effect of genotype (*p* = 0.29) or a genotype x time interaction (*p* = 0.95).

Tolerance to the antinociceptive effects of morphine was also assessed using the hotplate test. Similar to the tail-flick test, there was a significant effect of time on tolerance using the hotplate to assess antinociception (Fig 7B) (*F*_9,198_ = 31.28; *p*<0.0001). As with the tail-flick assay, there was no effect of genotype (p = 0.17) or a genotype x time interaction (*p* = 0.68).

Tolerance to the hypothermic effect of morphine was also assessed (Fig 7C). Data analysis revealed a significant effect of time (*F*_9,207_ = 44.07; *p*<0.0001) but not genotype (*p* = 0.12). Likewise, there was no genotype x time interaction (*p* = 0.96).

### Morphine dependence

Symptoms of naloxone-precipitated morphine withdrawal were measured to assess whether morphine dependence was changed in S426A/S430A mutant mice. There was no difference between genotypes in the number of morphine withdrawal-induced jumps (*p* = 0.26), diarrhea (*p* = 0.81), paw tremors (*p* = 0.41), and wet dog shakes (*p* = 0.71). There was a time dependent decrease in the number of jumps (*F*_11,132_ = 56.14; *p*<0.0001), diarrhea episodes (*F*_1,132_ = 6.468; *p*<0.0001), and paw tremors over the 60 minute observation period.

## Discussion

The endocannabinoid system has been shown to be an important regulator of ethanol- [11] and opioid- [12,13] motivated behavior. However, the effect of enhanced endocannabinoid signaling on ethanol and morphine behaviors is less well understood. This study was undertaken to investigate the hypothesis that enhanced CB_1_ signaling, caused by disruption of CB_1_ desensitization, potentiates opiate and alcohol addiction. Although ethanol intake and preference were modestly increased in a concentration-depedent mannner in the S426A/S430A mutant mouse model, no changes in ethanol reward, sensitivity, or metabolism were detected. There were also no differences in morphine reward or dependence.

A number of experiments were performed to ascertain the possible reason for the modest increase in consumption 6% ethanol in the S426A/S430A mutant mice. Preference for sucrose solution and aversion for quinine containing solutions were examined to determine whether taste preferences were altered in the mutant mice. However, no differences in taste preference were observed in S426A/S430A mutant mice. The possibility that increased drinking of 6% ethanol in the S426A/S430A mtuants was caused by an enhancement of the reinforcing effect of ethanol was assessed by testing ethanol CPP. Robust ethanol CPP (2 g/kg) that was equivalent in magnitude to what was observed in wild-type mice was found for the S426A/S430A mutant mice. Experiments assessing ethanol CPP were done using three chamber boxes as opposed to the two chamber boxes used for ethanol and morphine CPP. Thus a direct comparision of the time spent in the drug-paired (CS^+^) side between these two experiments is not possible due to the presence of the third chamber for the ethanol CPP experiment. This result demonstrates that reward for ethanol is normal and intact in the S426A/S430A mutants and suggests that altered endocannabinoid signaling in the reward circuitry (dopaminergic projections from the ventral tegmental area to the nucleus accumbens) is probably not responsible for increased ethanol consumption in these mutants. Finally, metabolism of a bolus injection of 2 g/kg ethanol was measured to determine whether increased or more rapid ethanol metabolism might underlie the modest increase in ethanol intake and preference observed in the S426A/S430A mutants. There was a significant but slight effect of genotype with wild-type mice showing slightly elevated BECs overall, there was no difference in BECs between wild-type and S426A/S430A mutants detected at 15, 30, 60 or 120 minutes following ethanol injection, suggesting that ethanol metabolism is not changed for S426A/S430A mutant mice. Thus, our results show an increase in ethanol consumption and preference for dilute ethanol solutions in S426A/S430A mutants that are not due to changes in reward, sensitivity, or metabolism for ethanol.

All of our work was done using only male mice. Given that female mice drink significantly more ethanol than their male counterparts in a 24 hour access free drinking paradigm similar to the one used in our study [42,43] it is possible that we might have seen greater effects of the S426A/S430A mutation on ethanol consumption and CPP had we used female mice instead male mice. For example, when looking at the role of the CB_1_ receptor in mediating ethanol intake, Hungund and colleages found that the effect of knocking out the CB_1_ receptor caused a much more subtle decrease in ethanol intake in male versus female mice [19]. This sex difference was driven entirely by the fact that female wild-type mice drank more than twice the ethanol (10 g/kg/day) than male wild-type mice (5 g/kg/day) resulting in a floor effect. Therefore, it would be interesting to determine whether the S426A/S430A mutation in females results in a greater difference in ethanol intake than we saw in males. Likewise, given that female mice were able to discriminate increasing concentration of ethanol (3 vs 10%) whereas male mice did not [43], it would be interesting to observe whether the S426A/S430A mutation alters the rewarding effects for ethanol CPP at 1 and 2 g/kg of ethanol in female mice.

Morphine-induced CPP and intravenous self-administration are completely absent in mice lacking CB_1_ receptors [24–26]. However, CPP for cocaine is not altered in CB_1_ knock-out (KO) mice [24]. Pharmacological blockade of CB_1_ using SR141617A (Rimonabant) attenuated CPP for morphine [44,45]. Direct activation of cannabinoid receptors using WIN 55,212–2 or URB597, an inhibitor of fatty acid amide hydrolase (FAAH), does not impact morphine CPP [46]. However, less is known about the effects of CB_1_ activation on morphine CPP. Therefore, S426A/S430A mutant mice were used to test whether enhanced endocannabinoid signaling, caused by blocking CB_1_ desensititization, might potentiate morphine CPP. Consistent with previous work using URB597 to potentiate endocannabinoid signaling [46], there was no evidence of increased morphine CPP in S426A/S430A mutant mice.

Previous work demonstrates evidence of bi-directional cross-tolerance between opioid and cannabinoid agonists [47,48]. Recent work demonstrated tolerance for WIN 55,212–2, CP55,940, and ∆^9^-THC in morphine-tolerant rhesus monkeys [49]. Tolerance to ∆^9^-THC was delayed in mice lacking pre-proenkephalin, the precursor for the opioid peptide enkephalin [50]. Chronic treatment of rodents with either ∆^9^-THC or CP55,940 causes cross-tolerance to morphine analgesia [48,51]. While co-administration of AM251, a CB_1_ inverse agonist, inhibited tolerance to the antinociceptive effects of chronic morphine [52]. Activation of CB_1_ with ∆^9^-THC [53] or CP55,940 [54] attenuated tolerance to the antinociceptive effects of morphine. Additional work found that chronic morphine treatment produced a large reduction of 2-AG levels in the brain, raising the possibility that decreased 2-AG might be involved in the development of morphine tolerance [51].

Therefore, tolerance to morphine was examined in S426A/S430A mutants based on strong evidence of interplay between the opioid and endocannabinoid systems and bi-directional cross-tolerance for agonists of these receptors. Tolerance for the antinociceptive effects of morphine in either the tail-flick or hotplate assay did not differ between S426A/S430A mutants and wild-type mice. This finding contrasts slightly with previous studies demonstrating that cannabinoid receptor activation can attenuate or prevent tolerance for morphine [53,54]. However, previous studies looked at how co-administration of CB_1_ agonists with morphine could reduce morphine tolerance [53,54], whereas the current study only looked at how morphine tolerance differed as a function of our desensitization-resistant S426A/S430A mutation. In addition, it is worth noting that constitutive activation of our S426A/S430A knock in mutation might result in subtle neuroadaptive changes within the endocannabinoid system that could mask subtle effects on morphine tolerance.

Treatment of rats or mice with rimonabant has been shown to reduce opioid dependence, suggesting a key role for CB_1_-mediated signaling in this phenomenon [28,55]. In addition, mice lacking CB_1_ receptors exhibit reduced morphine dependence [25,27,29]. However, increasing endocannabinoid levels using inhibitors of endocannabinoid catabolic enzymes also attenuates symptoms of precipitated withdrawal in morphine-tolerant mice [56,57]. Therefore, we wanted to test whether physical signs of morphine dependence were altered in S426A/S430A mutants. S426A/S430A mutants displayed no change in the number of morphine withdrawal-induced jumps, diarrhea episodes, paw tremors, or wet dog shakes compared to wild-type controls. This suggests that the modest enhancements in endocannabinoid signaling observed in the S426A/S430A mutants are not sufficient to alter morphine withdrawal.

Although a previous examination did not find altered endocannabinoid levels or CB_1_ protein in the brains of S426A/S430A mice under baseline conditions, we did not exhaustively examine these ECS components in all parts of the brain associated with drug taking behaviors and dependence [39]. Nor did we examine endocannabinoid or CB_1_ levels in mice given either morphine or ethanol or mice allowed to voluntarily drink ethanol. Thus, it is possible that homeostatic regulation of endocannabinoid signaling might restore normal balance and endocannabinoid tone in parts of the brain responsible for ethanol- and morphine-motivated behaviors. For example, it is possible that CB_1_ and endocannabinoid levels might be down-regulated in regions of the brain associated with morphine physical dependence such as the amygdala and noradrenergic neurons in the hindbrain. In a separate study, we did not observe evidence of metabolic abnormalities in S426A/S430A mutant compared to wild-type controls fed a routine or high fat diet [58]. One possible explanation for the lack of phenotype observed in these mutants could be that the S426A/S430A mutation is not sufficient to confer significantly enhanced responses to relatively low basal levels of endocannabinoids in tissues or circulation. Therefore, we conclude that this model might have limited utility for studying the effects of enhanced ECS signaling.

This work is timely given the prevalence and legalization (in some states) of recreational cannabis consumption, which represents a major source of excess ECS activation in humans. Additionally, expression of endocannabinoids in circulation and in tissue are increased for a number of disease states including obesity, cancer, and neurodegeneration. Thus, understanding the potential impact of elevated ECS signaling on developing dependence to drugs of abuse such as morphine and ethanol has tremendous relevance for human health. Although we observe increased ethanol drinking in the S426A/S430A mutants our results suggest that modest increases in endocannabinoid signaling, such as those that occur in these mutants, do not broadly impact drug reward and dependence.

## Supporting information

S1 Fig Daily consumption of 15% ethanol. (TIF)

S2 Fig Daily preference for 15% ethanol. (TIF)
